# Relationship between pain relief, reduction in pain-associated sleep interference, and overall impression of improvement in patients with postherpetic neuralgia treated with extended-release gabapentin

**DOI:** 10.1186/s12955-016-0456-0

**Published:** 2016-04-01

**Authors:** Neel Mehta, Iwona Bucior, Shay Bujanover, Rajiv Shah, Amitabh Gulati

**Affiliations:** Weill Cornell Medical College of Cornell University, New York, NY USA; Depomed, Inc., Newark, CA USA; St. Francis Hospital, Roslyn, NY USA; Memorial Sloan Kettering Cancer Center, New York, NY USA

**Keywords:** Neuropathic pain, Sleep, Quality of life, Postherpetic neuralgia, Gabapentin, Gastroretentive

## Abstract

**Background:**

Postherpetic neuralgia (PHN) interferes with patients’ quality of life, and disturbed sleep is a prevalent complaint. Pain-associated sleep interference in turn enhances pain and/or reduces pain tolerance. Therefore, reducing sleep interference by pain, in addition to pain control, may improve patient care. To address this notion, we characterized relationships among changes in pain intensity, sleep interference, and overall impression of improvement in PHN patients treated with gastroretentive gabapentin (G-GR).

**Methods:**

Patients with PHN (*n* = 556) received G-GR 1800 mg once-daily in two phase 3 and one phase 4 study. Visual Analog Scale (VAS) and Brief Pain Inventory (BPI) were completed at baseline and the end of study. Patients’ Global Impression of Change (PGIC) was completed at the end of study. Regression analyses examined relationships between VAS, BPI sleep interference by pain, and PGIC.

**Results:**

At the end of treatment, 53.7 and 63.2 % of patients reported a ≥30 % reduction in VAS and BPI pain-associated sleep interference (BPISI) respectively; 46.3 % reported feeling “Much” or “Very Much” improved on the PGIC. There were positive correlations between the percent reductions in VAS and BPISI; both correlated with PGIC improvements. Percent changes in VAS and BPISI were significant (*p* < 0.0001 and *p* = 0.0082, respectively), and were independent predictors of feeling “Much” or “Very Much” improved on the PGIC.

**Conclusions:**

Reductions in pain intensity and in BPISI were correlated, and both also correlated with overall impression of improvement for patients with PHN treated with G-GR. Both pain relief and improvement BPISI independently predicted improvement in PGIC. For optimal patient care, clinicians should consider reducing the impact of pain on quality of sleep as well as overall pain reduction.

**Trial registration:**

ClinicalTrials.gov numbers, NCT00335933, NCT00636636, NCT01426230.

## Background

Postherpetic neuralgia (PHN) is a chronic neuropathic pain syndrome resulting from nerve damage caused by the varicella zoster virus that is reactivated during acute herpes zoster (HZ, shingles) [1]. PHN, which occurs in up to 20 % of HZ patients, can be debilitating and interferes with patients’ physical function and their quality of life [2–6]. Consistent with the tendency of neuropathic pain to be worst during the night [7], sleep disturbance is one of the most common complaints among patients with PHN. Sleep disturbance may in turn lead to additional comorbid conditions such as anxiety or depression [5, 8–10], and some studies suggest that shortened or disturbed sleep may lead to reduced pain tolerance [11, 12]. Evidence supports a reciprocal relationship between pain and sleep in which pain disturbs sleep, and poor sleep enhances pain [6, 8, 13]. Thus, it is expected that improvement in sleep quality, in addition to control of neuropathic pain, may improve patients’ overall quality of life [14].

Although the beneficial effect of various formulations of gabapentin [G-GR [15, 16], gabapentin enacarbil [17], and an immediate-release gabapentin [18, 19] on the quality of sleep has been described in several studies, the relationship between changes in pain and sleep and how they contribute to overall patient improvement is complex, and are not well understood. A recent analysis of integrated data from phase 3 and 4 studies of gastroretentive gabapentin (G-GR) 1800 mg once-daily reported widespread, networked, positive correlations among efficacy endpoints, including among pain qualities on the VAS and BPI, pain interference on the BPI, and overall improvement on the PGIC [20]. In the current study, we extend these findings by examining, at the individual patient level, the relationship between changes in pain intensity and pain interference with sleep, and how changes in these measures contribute to patient’s overall impression of improvement.

## Methods

### Patients

Individual patient data from 566 PHN patients in two double-blind, randomized, placebo-controlled phase 3 studies (NCT00335933 and NCT 00636636) and one open-label, single-arm phase 4 study (NCT01426230) were pooled in the analysis. Patient inclusion and exclusion criteria of individual studies included in this analysis have been described in detail elsewhere [21–23]. Briefly, in the phase 3 studies, eligible patients were ≥18 years, with neuropathic pain for ≥3 months or ≥6 months after the healing of herpes zoster skin rash, and had an average daily pain score of ≥4 based on an 11-point Likert scale (where 0 = no pain and 10 = worst possible pain). In the phase 4 study, patients were relatively unselected to best reflect the real-world population, and included patients ≥18 years with active PHN, regardless of their baseline pain scores. Only patients with valid baseline efficacy measures and who received treatment with G-GR 1800 mg once daily were included. Individual study protocols were approved by appropriate institutional review boards/ethics committees for each center and were conducted in accordance with International Conference on Harmonization (ICH) Good Clinical Practice guidelines. Written informed consent was obtained from each patient prior to enrollment.

### Treatments

All three studies shared a similar G-GR treatment schedule: a 2-week titration period, a stable dose treatment period (8 weeks for phase 3, and 6 weeks for phase 4), and a 1-week dose tapering period. The 2-week titration period used a set schedule: Day 1: 300 mg; Day 2: 600 mg; Days 3–6: 900 mg; Days 7–10: 1200 mg; Days 11–14: 1500 mg; Day 15: 1800 mg.

### Efficacy evaluations

In the current analysis, pain intensity scores were from the 100-mm VAS, which was used in all three studies. For the phase 3 studies, VAS was a component of the Short-Form-McGill Pain Questionnaire (SF-MPQ), completed as a secondary efficacy variable. In the phase 4 study, VAS was the primary efficacy variable for measurement of pain intensity. Pain interference with sleep was evaluated using the BPI, which was one of the secondary efficacy endpoints in both the phase 3 and phase4 studies. Pain-associated BPI sleep interference (BPISI) was assessed on an 11-point numeric rating scale (NRS) ranging from 0 (pain does not interfere with sleep) to 10 (pain completely interferes with sleep). Overall improvements on the PGIC were evaluated as secondary efficacy endpoints in phase 3 and phase 4 studies. The VAS and BPI were completed at the end of the baseline week, at Week 2, and at the end of the efficacy treatment period (Week 8 or 10) or early termination. The PGIC was completed at the end of the efficacy treatment period (Week 8 or 10) or early termination. For the integrated analysis, the end of the study was defined as Week 10 for phase 3, and Week 8 for phase 4.

### Statistical methods

Efficacy analyses were performed for all patients who received ≥1 dose of study drug. Percent changes from baseline to the end of the study in VAS pain intensity and BPISI, and the proportion of patients categorized as “Very Much Improved”, “Much Improved”, “Minimally Improved”, “No Change”, “Minimally Worse”, “Much Worse”, or “Very Much Worse” on the PGIC at the end of the study were determined. Changes from baseline in VAS and BPISI scores were estimated with an analysis of covariance (ANCOVA) model that included treatment, study centers, and the baseline value as covariates. As last observation carried forward (LOCF) was the pre-determined method approved for all individual studies, missing data were imputed by LOCF to follow approved protocols. Exploratory analyses were designed to examine relationships among treatment outcomes for patients with potentially clinically significant responses to treatment with G-GR. Therefore, “Very Much Improved” and “Much Improved” responses on the PGIC were grouped together, and “Minimally Improved”, “No Change”, “Minimally Worse”, “Much Worse”, or “Very Much Worse” were grouped into “Not Improved”. Furthermore, in accordance with the published literature and the consensus summary statement produced by the Initiative on Methods, Measurement, and Pain Assessment in Clinical Trials (IMMPACT) [24–26], reductions of ≥30 % served as determinants of clinically important reductions from baseline in the VAS or BPISI scores. Relationships between percent changes in various efficacy outcomes were examined using linear regression model ANOVA. Multivariable logistic regression analyses were performed to evaluate percent changes in VAS and BPISI as predictive factors for being “Much” or “Very Much” improved on the PGIC. To measure the degree of linear dependence between percent reductions in the VAS and BPISI scores, the Pearson correlation coefficient (r) was determined.

## Results

### Patient characteristics

The integrated dataset from the phase 3 and 4 studies included 546 patients in the efficacy population and 556 patients in the safety population. Patient demographics and baseline disease characteristics were similar between studies (Table 1). The mean patient age was 66.7 years, and the majority of patients were Caucasian (86.2 %) and female (60.3 %). The mean baseline VAS score was 66.1 mm on the 100-mm scale. The mean baseline BPISI score was 5.1 on the 0–10 NRS scale, and 9 % of patients had a baseline score of 0.

**Table 1:** Patient demographics and baseline disease characteristics, safety population

	G-GR 1800 mg/day
	(*n* = 556)
Age (years)	
Mean (SD)	66.7 (12.9)
Median	69.0
Range	18–92
Age category, n (%)	
< 55 years	89 (16.0)
55–64 years	109 (19.6)
65–74 years	195 (35.1)
≥ 75 years	163 (29.3)
Sex, n (%)	
Male	221 (39.7)
Female	335 (60.3)
Race, n (%)	
Caucasian	479 (86.2)
African American	29 (5.2)
Hispanic	38 (6.8)
Other	10 (1.8)
Baseline VAS (mm)	
Mean (SD)	66.1 (56.9)
Median	64.0
Range	2–91
Baseline VAS category, n (%)	
≤ 20 mm	15 (2.7)
> 20 mm	531 (95.5)
Baseline BPI Sleep Interference by Pain	
Mean (SD)	5.1 (2.9)
Median	5
Range	0–10

### Correlation between pain relief and sleep interference by pain improvement

**Fig. 1:**
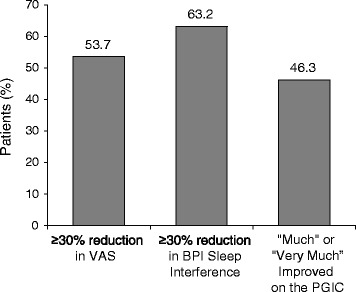
Proportion of patients who reported ≥30 % reductions in the VAS or BPI Sleep Interference by Pain scores, or who reported feeling “Much” or “Very Much” improved at the end of G-GR treatment

**Fig. 2:**
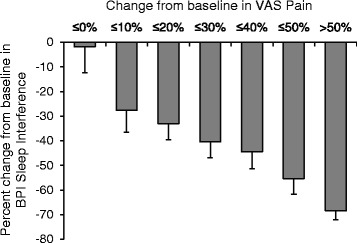
Percent reduction from baseline in the BPISI score by categories of percent reduction from baseline in the VAS score

At the end of G-GR treatment, 53.7 % of patients in the efficacy population reported ≥30 % reduction in pain intensity on the VAS, 63.2 % of patients reported ≥30 % reduction in BPISI, and 46.3 % of patients reported feeling “Much” or “Very Much” improved on the PGIC (Fig. 1). A comparison of changes in VAS and BPISI scores from baseline showed that a larger percent reduction from baseline in the VAS score was associated with a larger percent reduction in the BPISI score at the end of the study (Fig. 2). For patients with clinically significant reduction (defined as ≥30 % reduction from baseline) in the VAS score, there was a linear correlation between percent reductions in the VAS and BPISI scores, (*r* = 0.28, *p* < 0.0001), whereas for patients with <30 % reduction in the VAS score, there was no linear correlation with BPISI (*r* = 0.0612, *p* = 0.3608; Table 2). Significantly more patients with ≥30 % reduction in the VAS score from baseline than with <30 % reduction in the VAS score (72.0 vs. 39.5 % of patients, *p* < 0.0001) simultaneously reported ≥30 % reduction in the BPISI score (Table 3). Also, among patients with ≥30 % reduction in the VAS score from baseline, more simultaneously reported ≥30 % reduction in the BPISI score than <30 % reductions in BPISI score (72.0 vs. 19.1 % of patients, *p* < 0.0001).

**Table 2:** Linear Pearson correlation coefficient correlation between VAS and BPI sleep interference by pain

	n	Pearson correlation coefficient (r)	*p*-value
≥30 % reduction in VAS	267	0.2795	<0.0001
<30 % reduction in VAS	225	0.0612	0.3608

**Table 3:** Relationship between percent reduction from baseline in BPI sleep interference by pain and percent reduction in VAS

	≥30 % reduction in VAS^a^	<30 % reduction in VAS^a^	Difference	*p*-value
	(*n* = 293)	(*n* = 253)	% (95 % CI)	
≥30 % reduction in BPI Sleep Interference, *n* (%)	211 (72.0)	100 (39.5)	32.49 (24.57, 40.41)	<0.0001
<30 % reduction in BPI Sleep Interference, *n* (%)	56 (19.1)	125 (49.4)	−30.29 (−37.93, −22.66)	<0.0001
Difference, % (95 % CI)	52.90 (46.07, 59.73)	−9.88 (−18.50, −1.26)	n/a	n/a
*p*-value	<0.0001	0.0253	n/a	n/a

^a^Missing data were excluded; number of patients does not add up to 100 %; *n/a*, not applicable

### Influence of pain relief and sleep improvement on overall impression of improvement

**Fig. 3:**
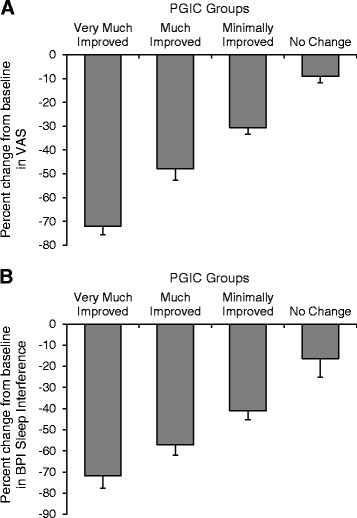
Percent reduction from baseline in the VAS (**a**) and BPISI (**b**) scores by categories of improvement on the PGIC

Better overall improvement as assessed by the PGIC was associated with larger percent reductions from baseline in the VAS (Fig. 3a) and BPISI (Fig. 3b) scores. Significantly more patients with ≥30 % reduction than with <30 % reduction in the VAS score (70.2 vs. 17.9 % of patients, *p* < 0.0001) were “Much” or “Very Much” improved on the PGIC (Table 4). Furthermore, among patients with ≥30 % reduction in the VAS score, more were “Much” or “Very Much” improved on the PGIC than not improved (70.2 vs. 29.8 % of patients, *p* < 0.0001). In contrast, among patients with <30 % reduction in the VAS score, more were not improved than “Much” or “Very Much” improved on the PGIC (82.1 vs. 17.9 % of patients, *p* < 0.0001). The relationship between percent reduction in the BPISI score and improvements on the PGIC was similar, and more patients with ≥30 % reduction than with <30 % reduction in the BPISI score (57.8 vs. 26.9 % of patients, *p* < 0.0001) were simultaneously “Much” or “Very Much” improved on the PGIC (Table 5).

**Table 4:** Relationship between percent reduction from baseline in VAS and improvement on the PGIC

	≥30 % reduction in VAS	<30 % reduction in VAS	Difference	*p*-value
	(*n* = 285)	(*n* = 240)	% (95 % CI)	
“Much” or “Very Much” improved on PGIC, n (%)	200 (70.2)	43 (17.9)	52.26 (45.06, 59.45)	<0.0001
Not improved on PGIC^a^, n (%)	85 (29.8)	197 (82.1)	−52.26 (−59.45, −45.06)	<0.0001
Difference, % (95 % CI)	40.35 (32.84, 47.86)	−64.17 (−71.03, −57.31)	n/a	n/a
*p*-value	<0.0001	<0.0001	n/a	n/a

^a^Includes patients from other PGIC categories: Minimally Improved, No Change, Minimally Worse, Much Worse, Very Much Worse; *n/a*, not applicable

**Table 5:** Relationship between percent reduction from baseline in BPI Sleep Interference by Pain and improvement on the PGIC

	≥30 % reduction in BPI Sleep Interference	<30 % reduction in BPI Sleep Interference	Difference	*p*-value
	(*n* = 301)	(*n* = 171)	% (95 % CI)	
“Much” or “Very Much” improved on PGIC, *n* (%)	174 (57.8)	46 (26.9)	30.91 (22.23, 39.58)	<0.0001
Not improved on PGIC^a^, *n* (%)	127 (42.2)	125 (73.1)	−30.91 (−39.58, −22.23)	<0.0001
Difference, % (95 % CI)	15.61 (7.72, 23.50)	−46.29 (−55.60, −36.80)	n/a	n/a
*p*-value	0.0001	<0.0001	n/a	n/a

^a^Includes patients from other PGIC categories: Minimally Improved, No Change, Minimally Worse, Much Worse, Very Much Worse; *n/a*, not applicable

**Fig. 4:**
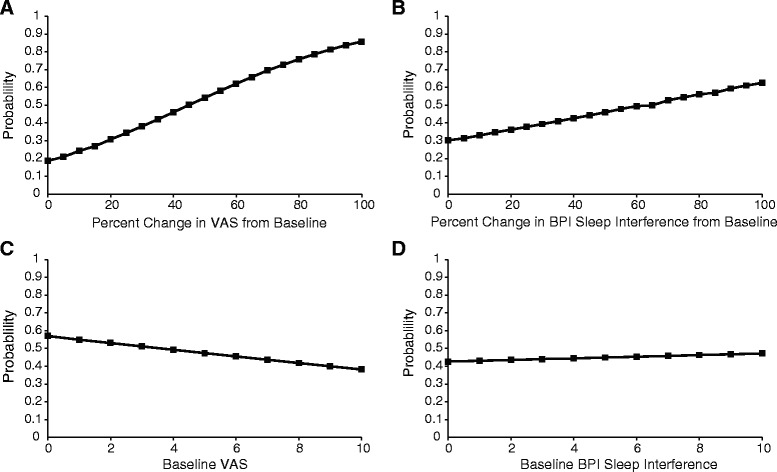
Influence of the VAS and BPI Sleep Interference by Pain scores on the probability to be “Much” or “Very Much” improved on the PGIC. Probabilities to be “Much” or “Very Much” improved on the PGIC were determined by multiple logistic regression analyses for percent changes from baseline in the VAS score (**a**) and BPI Sleep Interference by Pain score (**b**), and for baseline VAS (**c**) and BPI Sleep Interference by Pain (**d**) scores

Additional exploratory analyses were done to evaluate the influence of percent changes in the VAS and BPI Interference scores on being “Much” or “Very Much” improved on the PGIC. The probability of being “Much” or “Very Much” improved on the PGIC increased with greater percent reductions from baseline in the VAS (Fig. 4a) and BPISI (Fig. 4b) scores, whereas baseline values did not have a positive effect (Figs. 4c, d). Overall, percent changes in the VAS score had a greater influence on the probability of being “Much” or “Very Much” improved on the PGIC than did percent changes in the BPISI score (*p* < 0.0001 vs. *p* = 0.0063). Baseline VAS values had a marginally significant (*p* = 0.0429) negative effect, and baseline BPISI values did not have a significant influence (*p* = 0.8458). Furthermore, percent changes from baseline in VAS and BPISI scores were both significant predictive factors for being “Much” or “Very Much” improved on the PGIC (Table 6). However, compared with the BPISI, the percent change in VAS had a larger regression coefficient (0.03 vs. 0.006) and was of greater significance (*p* < 0.0001 vs. *p* = 0.0082). Because adding the interaction between percent changes in the VAS and BPISI scores to the regression model showed no significance (*p* = 0.4808) for being “Much” or “Very Much” improved on the PGIC (Table 6), these variables acted as independent predictive factors for overall improvement.

**Table 6:** Comparison between percent changes in VAS and BPI Sleep Interference by Pain as predictive factors for being “Much” or “Very Much” improved on the PGIC

Dependent variable	Predictive factors	Regression coefficient	*p*-value
“Much” and “Very Much” improved on the PGIC	Percent change in VAS	0.02988	<0.0001
	Percent change in BPI Sleep Interference	0.00616	0.0082
	Interaction between percent changes in VAS and BPI Sleep Interference	0.00005	0.4808

## Discussion

The intensity of neuropathic pain tends to progress throughout the day, being worst at night and significantly impairing sleep [6, 7, 27]. Inadequate or poor-quality sleep may in turn contribute to stress and other negative consequences of living with pain, including reduced pain tolerance [6, 8, 10, 13]. In this analysis, we investigated the correlation between G-GR-mediated changes in BPISI, VAS pain scores, and PGIC scores at the individual patient level.

In a prior analysis, positive correlations were observed among VAS, overall BPI scores, pain interference on the BPI, and PGIC, suggesting positive feedback loops in which pain interferes with patient functioning, and poor functioning enhances pain [20]. Here we have extended the analysis to evaluate the relationship with pain-associated sleep interference. As in our prior analysis, a 30 % reduction in pain intensity was chosen as a cut-point for evaluating correlations. In accordance with IMMPACT recommendations for determining clinically important differences in pain intensity reductions ≥30 % are considered “moderately important” improvements, whereas decreases of ≥50 % are considered “substantial” improvements [26, 28]. Use of the 30 % cutoff was considered a conservative approach.

Not surprisingly, clear correlations were observed in our analysis among pain reduction, pain-associated sleep interference and PGIC. Consistent with the notion that mechanisms underlying pain relief and sleep improvement may be distinct, both VAS and BPISI were independent predictors for patients’ reporting “Much” or “Very Much” improvement on the PGIC. In addition, 39.5 % of PHN patients with no clinically significant reduction in VAS still had a clinically significant reduction in BPISI. As gabapentin is known to improve sleep quality by increasing slow-wave sleep in both normal adults (31) and in patients with epilepsy (32), a direct effect on sleep may have contributed to improvements in BPISI independent of pain reduction.

No correlations were observed between BPISI and VAS for those with a ≤30 % change. This result may not be surprising, since a < 30 % change in VAS is considered minimally important; it is likely that any correlations, should they exist, are lost due to the very low signal.

It is important to note that approximately 22 % of patients did not report clinically significant reductions in pain or BPISI but still reported feeling “Much” or “Very Much” improved on the PGIC at the end of treatment. These results suggest a complex relationship among patient-reported outcomes and that changes in other efficacy measures, such as mood, opioid usage, and return to work may also play a role. An analysis of pain reduction, pain interference with sleep, and PGIC in patients treated with placebo may also be of interest. In this study, since the phase 4 study was a real-world, open label study with no placebo control group, a rigorous analysis was not possible.

Our results are consistent with a study of pregabalin in patients with diabetic peripheral neuropathy (DPN) or PHN that showed similar correlations between pain relief, sleep improvement, and improvements in quality of life, and these improvements were not solely mediated via control of pain or sleep disturbance [14]. Interestingly, a different pregabalin study in patients with neuropathic pain of any origin reported that improvement in sleep was a better predictor than a reduction in pain intensity of improvements in health-related quality of life [29].

## Conclusions

For the majority of patients with PHN and treated with G-GR in these clinical trials, clinically significant reductions in pain intensity and pain-associated sleep interference were correlated with, and independently predicted, feeling “Much” or “Very Much” improved at the end of the G-GR treatment. For optimal patient care, clinicians should consider reducing the impact of pain on quality of sleep as well as overall pain reduction.
